# Heterogeneous nuclear ribonucleoprotein K: altered pattern of expression associated with diagnosis and prognosis of prostate cancer

**DOI:** 10.1038/sj.bjc.6605057

**Published:** 2009-04-28

**Authors:** P Barboro, E Repaci, A Rubagotti, S Salvi, S Boccardo, B Spina, M Truini, C Introini, P Puppo, N Ferrari, G Carmignani, F Boccardo, C Balbi

**Affiliations:** 1 Istituto Nazionale per la Ricerca sul Cancro, Largo Rosanna Benzi, 10-16132 Genova, Italy; 2 Dipartimento di Oncologia, Biologia e Genetica, Università di Genova, Largo Rosanna Benzi, 10–16132 Genova, Italy; 3 Dipartimento di Urologia Università di Genova, Largo Rosanna Benzi, 10–16132 Genova, Italy

**Keywords:** heterogeneous nuclear ribonucleoprotein K, prostate cancer, nuclear matrix, immunohistochemistry, western blot, biological markers

## Abstract

Using proteomic analysis of the nuclear matrix (NM), we found that heterogeneous nuclear ribonucleoprotein K (hnRNP K), a member of the hnRNP family with pleiotropic functions, was differentially expressed in prostate cancer (PCa) tissues. This study aimed to characterise the expression of hnRNP K and its subcellular localisation in PCa, utilising immunohistochemical and quantitative western blot techniques. Furthermore, the hnRNP K expression was studied in human PCa cell lines in order to determine its modulation by bicalutamide, the anti-androgen widely used in PCa therapy. Immunohistochemical staining of paraffin-embedded tissues showed that hnRNP K was overexpressed in PCa, where it was localised both in the cytoplasm and in the nucleus. Staining of non-tumour tissues showed exclusively nuclear localisation and a less intense or absent signal. Immunoblot analysis demonstrated that the hnRNP K level within the NM was higher in PCa compared with non-tumour tissues and closely correlated with Gleason score (*P*=0.008). Higher expression within the NM was significantly (*P*=0.032) associated with poor prognosis. In two-dimensional western blot analysis hnRNP K presented several isoforms; the one with pI 5.1 was the most differently expressed between non-tumour and PCa tissues. Preliminary results indicate that hnRNP K can be modulated *in vitro* by a non-steroidal anti-androgen. Taken together, our findings suggest that hnRNP K has potential implications at the diagnostic, prognostic and therapeutic levels in PCa.

Prostate cancer (PCa) continues to represent a major health concern. As the introduction of serum prostate-specific antigen (PSA) into clinical practice in the late 1980s, the incidence of this tumour has increased whereas the impact on mortality rates is less clear-cut. PSA has poor specificity and cannot distinguish indolent tumours from the aggressive ones that need immediate treatment. New investigation into more accurate diagnostic and prognostic biomarkers is needed to improve risk stratification and identify new targets for therapy of PCa (Damber and Aus, 2008).

As the nuclear matrix (NM) is considered to be a good source of cancer-specific biomarkers (Leman and Getzenberg, 2008), we utilised a proteomic approach to compare the NM proteins of PCa with those isolated from non-tumour (NT) tissues. In PCa, we observed an increase in the complexity of the protein pattern, which correlated with the level of tumour differentiation (Alberti *et al*, 1996, 2000); moreover, a few proteins (called NM-6, 7 and 8) were significantly correlated with the risk of biochemical progression (Boccardo *et al*, 2003). To characterise these potential biomarkers, protein spots were selected from high-resolution two-dimensional gels and analysed by mass spectrometry. NM-6 was identified as heterogeneous nuclear ribonucleoprotein K (hnRNP K) (Barboro *et al*, 2005).

HnRNP K is a member of the large hnRNP family. It is a ubiquitous protein present primarily in the nucleus, but it has also been found in the cytoplasm and mitochondria; it is involved in the transcription, splicing and translation processes (Bomsztyk *et al*, 2004). It is active at the chromatin level, where it is present in a higher density at transcribed genes with respect to silent ones (Ostrowski *et al*, 2003). Moreover, hnRNP K binds directly to the promoter region of the human c*-myc* gene (Michelotti *et al*, 1996) and promotes neoplastic transformation in an eIF4E-dependent manner (Lynch *et al*, 2005). In breast cancer cells, overexpression of hnRNP K enhances cell proliferation and anchorage-independent growth (Mandal *et al*, 2001), and in several states of enhanced cell proliferation, increased expression of this protein has also been found (Ostrowski and Bomsztyk, 2003). Overexpression of hnRNP K has been shown in many human tumours too, including lung cancer (Pino *et al*, 2003), esophageal cancer (Hatakeyama *et al*, 2006), oral squamous cell carcinoma (Roychoudhury and Chaudhuri, 2007), colorectal cancer (Carpenter *et al*, 2006b) and nasopharyngeal carcinoma (Chen *et al*, 2008). Interestingly, in the latter three both aberrant hnRNP K localisation and a correlation between protein expression and patient's prognosis were also observed. Until now, very little is known about the behaviour of hnRNP K in PCa; Nagano *et al* (2004) demonstrated that the hnRNP K was strongly overexpressed in PCa with respect to normal cell lines derived from the same patient and that the protein was differentially modulated in two cell lines by INF. More recently, Wang *et al* (2008) have shown that a novel transcriptional repressor complex containing Pur*α* and hnRNP K binds the androgen receptor (AR) gene both in cell lines and in human prostate tissues. These results prompted us to better characterise the role of this protein in PCa.

In this study, we have examined the expression of hnRNP K in both samples from PCa tissues and cultured cell lines. We have analysed whether the alterations of this protein are correlated with clinicopathological characteristics and with the follow-up of patients. In addition, we have studied the sensitivity of hnRNP K to anti-androgen treatment. Our findings strongly suggest that hnRNP K is involved in the carcinogenesis process in PCa, is a potential diagnostic and prognostic marker and could be used to monitor therapeutic efficiency of anti-androgen agents.

## Materials and methods

### Patients and tissue samples

Studies were performed on PCa specimens obtained from 49 patients undergoing radical retropubic prostatectomy for clinically localised PCa between 1996 and 2003. NT tissue was obtained from contralateral lobe to the cancer zone and four normal human prostates (NHP) were collected from patients undergoing cystectomy for bladder cancer. The project was approved by the local Ethics Committee. Fresh tissues were immediately frozen in liquid nitrogen until sample preparation. All tissues were histologically confirmed by haematoxylin and eosin staining of frozen sections and only the specimens containing more than 80% of tumour cells were processed to isolate the NM. The patients’ characteristics are summarised in Table 1 and the tumours were classified according to the TNM system. Out of 49 patients included in the present analysis five patients received postoperative irradiation, five were treated with adjuvant hormone therapy and one with both treatments. Patients were followed at regular intervals and PSA determined. A PSA level of at least 0.4 ng ml^–1^, which was confirmed by another assay 4 weeks later, was sufficient to indicate a biochemical progression. After a median follow-up time of 49.9 months (95% confidence interval (CI) 20.4–80.9), 15 patients were found to have experienced biochemical progression.

### Cell culture

LNCaP and PC3 prostate carcinoma cell lines (ATCC, Rockville, MD, USA) were cultured in RPMI-1640 (Celbio, Milan, Italy) containing heat-inactivated 10% fetal bovine serum, 1% penicillin, 1% streptomycin and 1% glutamine. LNCaP medium was also supplemented with 10 mM HEPES, 1 mM sodium pyruvate and 4.5 mg/ml glucose. Cells were cultured in a monolayer in the presence of 0.1 nM 5-*α*-dihydrosterone for 72 h at 37°C in 5% CO_2_, and subsequently 10 *μ*M Bicalutamide (BIC) was added for a further 72 h.

### Cell fractionation

The NM was isolated both from tissues and cell lines as already described (Barboro *et al*, 1996) with minor modifications. The nuclear pellet was resuspended in digestion buffer consisting of 10 mM NaCl, 3 mM MgCl_2_, 10 mM Tris-HCl (pH 7.8) (all from Sigma, St. Louis, MO, USA) and 2 mM vanadyl ribonucleoside complex (New England BioLabs, Beverly, MA, USA), which was added to prevent the activation of endogenous RNase. Digestion by 1 U *μ*l^–1^ DNaseI RNase-free (Roche, Mannheim, Germany) was allowed to proceed for 1 h at room temperature. Chromatin fragments were extracted by addition of (NH_4_)_2_SO_4_ to a final concentration of 0.25 M. The NM was recovered by centrifugation at 10 000 **g** for 15 min and extracted again with a large excess of digestion buffer containing 0.25 M (NH_4_)_2_SO_4_. The sample was again pelleted and solubilised for one- or two-dimensional (1D- or 2D)-PAGE.

Cytoplasmic and nuclear extracts were prepared from cell lines exactly as reported by Kim and Tucker (2006). The nuclear fraction was then suspended in 400 *μ*l of 10% SDS, 3% dithiothreitol, 40 mM Tris and 0.1 mM EDTA, pH 6.1 and sonicated for 15 s on low power. SDS and dithiothreitol were added to the cytoplasmic fraction at final concentrations of 10 and 3%, respectively. Solubilisation of the nuclear and cytoplasmic fractions was carried out for 5 min at 100°C. The samples were delipidated and cleaned as described by Candiano *et al* (2004).

Protein concentrations were determined using the Bio-Rad (München, Germany) protein microassay with bovine serum albumin as a standard.

### Immunohistochemistry

Immunohistochemistry was carried out using an anti-hnRNP K antibody (sc-28380, Santa Cruz Biotechnology Inc., Santa Cruz, CA, USA) diluted 1:800. For each patient, both PCa and NT tissues were analysed and the more representative tumour sections were selected. Two different sections (3 *μ*m) of the same sample were treated independently. The sections were deparaffinised and immunostained using a BenchMark XT automated stainer (Ventana Medical Systems, SA Strasbourg, France). The antigen-antibody complex was revealed with the Ventana Medical System/View DAB (diaminobenzidine) detection system. The sections were then counterstained with Gill's haematoxylin and mounted in Eukitt. An appropriate positive tissue control was used for each staining run; the negative control consisted of performing the entire immunohistochemistry procedure on adjacent sections in the absence of the primary antibody. The sections were observed with an Olympus light microscope using × 10 and × 40 objectives. The extent of immunochemical reactivity in tumour and non-tumour cells were independently evaluated by two observers and graded according to the number of immunoreactive cells and staining intensity using the scoring system described by Carpenter *et al* (2006b).

### Gel electrophoresis

1D-PAGE was carried out according to Laemmli (1970). Eight μg of protein extracted from different cell fractions (cytoplasm, nucleus and NM) were loaded onto gels and separated at 5 mA/gel for 16 h at a constant temperature of 12°C.

High-resolution 2D-PAGE was performed as described earlier (Barboro *et al*, 2008). The gels were silver stained (Heukeshoven and Dernick, 1988) for protein pattern analysis or processed for western blotting. All of the silver-stained 2D gels were digitised with a GS-800 densitometer (Bio-Rad) using the same scanning conditions. Spot detection, gel alignment and normalisation were performed using the PDQuest software package (Ver. 7.3.0, Bio-Rad).

### Western blot analysis (WB)

Proteins separated by 1D- or 2D-PAGE were transferred to a Hybond-P membrane (Amersham Biosciences, Piscataway, NJ, USA) and immunodetection was carried out using the anti-hnRNP K antibody diluted 1 : 1200 (Santa Cruz Biotechnology Inc.). Peroxidase-conjugated anti-mouse (diluted 1 : 1000) was used as secondary antibody. As the major components of the NM undergo appreciable changes during PCa development, it was not possible to use an internal control to ensure equal loading on the gels. Therefore, the relative amounts of hnRNP K were determined following the quantitative method described by Alberti *et al* (2000). Briefly, equal quantities (8 *μ*g) of the same preparation were loaded on two 1D-PAGE gels and submitted to electrophoresis. One gel was stained with Coomassie brilliant blue R-250 and densitometric scans were performed in a GS-800 densitometer (Bio-Rad) and the total amounts of protein (*A*) were evaluated by integration of the optical density curve. The second gel was blotted and immunoreactive bands were detected using Hyperfilm ECL films (GE Healthcare, Piscataway, NJ, USA), which exhibit a linear response to light produced from enhanced chemiluminescence. The relative amounts of hnRNP K were obtained by normalising the integrated optical density by ECL (*B*) to the integrated optical density (*A*) of the corresponding Coomassie-stained gel. To reduce differences arising from fluctuations in the experimental conditions of the immunoblot, a fixed (8 *μ*g) amount of total protein extracted from PC3 from the same batch were run on the gels and the ratio *B/A* further normalised by the ECL signal of hnRNP K standard. This method allowed us to obtain quantitative results.

### Confocal laser scanning microscopy

LNCaP cells were grown on chamber slides. The slides were washed two times in PBS, fixed for 15 min in 3.7% formaldehyde and treated for 5 min with PBS containing 0.2% Triton X-100. After 15 min of blocking in PBS containing 2% BSA, cells were incubated for 30 min in the same buffer with mouse anti-hnRNP K antiserum (diluted 1:1000; Santa Cruz Biotechnology Inc.). The cells were then washed three times with PBS before a 30-min incubation with anti-mouse Alexa Fluor 633-conjugated immunoglobulin (diluted 1 : 500; Molecular Probes Inc., Eugene, OR, USA). To visualise nuclei, the cells were incubated with SYTOX Orange nucleic acid stain (diluted 1 : 10 000; Molecular Probes Inc.) for 10 min and washed in PBS. The cells were then mounted with 80% glycerol in PBS. Immunofluorescence staining was visualised using an Olympus FluoView CLSM-FV500 ( × 40 and × 60 objectives with an oil-immersion lens).

### Statistical analysis

Associations among the principal variables on study, that is, hnRNP K, age, preoperative PSA, Gleason score, capsular penetration, surgical margins, seminal vesicles involvement and pelvic nodes were investigated by using the Pearson correlation coefficient (Fleiss, 1981). To this aim, variables were categorised as follows: Gleason score (⩽7 *vs* >7), capsular penetration (not *vs* yes), surgical margins (not involved *vs* involved), seminal vesicles (not involved *vs* involved), pelvic nodes (not involved *vs* involved); hnRNP K, age and PSA were considered as continuous variables. Biochemical progression-free survival was defined as the time from surgery to PSA failure (PSA level of 0.4 ng ml^–1^). Curves were constructed using the Kaplan–Meier method and compared using the log-rank test. All of the *P*-values reported are two sided and *P*⩽0.05 was considered statistically significant (Armitage *et al*, 2000). The analyses were performed using SPSS version 15.0 for Windows.

The comparison of the relative amounts of hnRNP K was performed using *t*-test within the OriginPro 7.5 software, exporting the single value of each film.

## Results

### Expression and subcellular distribution of hnRNP K in PCa

**Figure 1:**
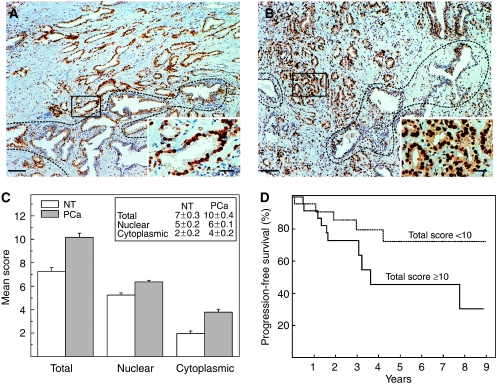
Immunohistochemical analysis of hnRNP K expression in PCa. (**A, B**) Representative images of two PCa samples with different total scores (9 and 14, respectively); higher magnification of the areas enclosed with lines are reported in the insets. Elevated hnRNP K staining was present both in the nucleus and cytoplasm of all tumour cells. The dash lines mark the NT areas. The bars correspond to 100 *μ*m in (**A**) and (**B**) and 25 μm in the insets. (**C**) Comparison of the scores of hnRNP K in NT and PCa tissues. The ordinates represent the mean score±s.e. (reported also in the inset); hnRNP K expression was significantly (*P*<0.0001) higher in PCa compared with NT tissue both in the cytoplasm and the nucleus. (**D**) Biochemical (PSA) progression-free survival according to hnRNP K expression. The dotted curve corresponds to patients (*n*=24) whose tumours had a low total score (<10) and the solid curve those (*n*=24) with high total scores (⩾10); *P*=0.085.

After the identification of the hnRNP K as a potential biomarker by proteomic analysis (Barboro *et al*, 2005) in PCa, we have examined its expression by immunohistochemical (IHC) staining. In NT tissue, hnRNP K was mostly localised in the nucleus and very low staining in the cytoplasm was observed; in PCa, the protein was overexpressed and localised both in the cytoplasm and in the nucleus (Figure 1A and B). No relationship was seen between hnRNP K staining and Ki-67 proliferation index (data not shown). The scores reported in Figure 1C demonstrate that hnRNP K expression was significantly higher (*P*<0.0001) in PCa with respect to NT tissue, both in the cytoplasm and the nucleus. Besides, more than 80% of the samples of PCa had a high score (⩾6) in the nucleus. These results thus confirm the potential diagnostic value of this protein. No significant correlation was found between both nuclear and cytoplasmic hnRNP K immunostaining and clinicopathological variables; however, the patients (*n*=24) with low total scores (<10) showed a trend towards better outcomes, which, however, was not statistically significant (*P*=0.085) (Figure 1D).

### Expression of hnRNP K associated with the NM in PCa

**Figure 2:**
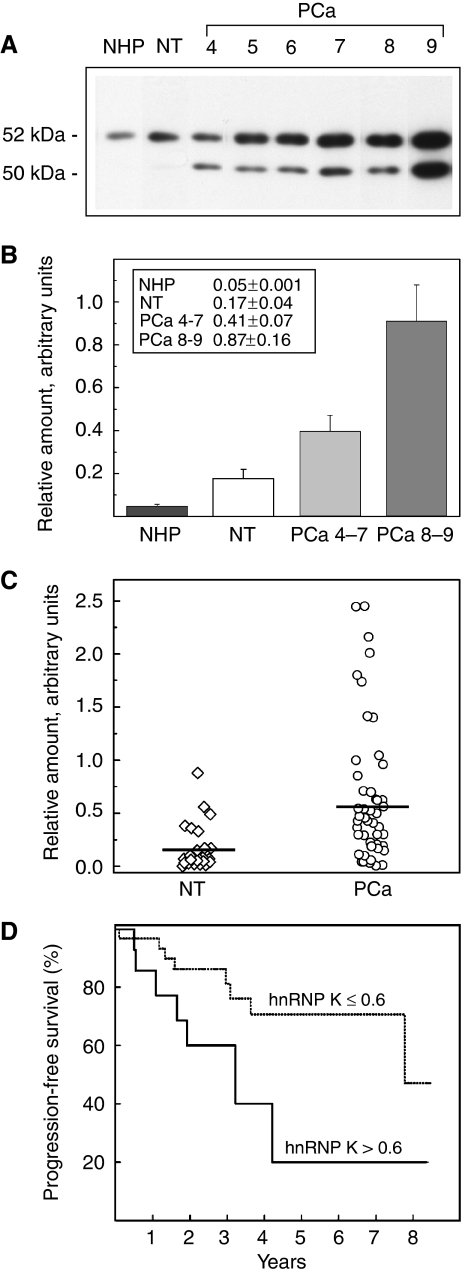
Expression of hnRNP K associated with NM in PCa. (**A**) Representative 1D WB analysis of NM isolated from NHP, NT and PCa with different Gleason scores. The relative molecular weights of hnRNP K and the splicing variants J are reported on the left. (**B**) The comparison among the relative amounts of hnRNP K as determined by quantitative analysis of four NHP, 24 NT and 27 PCa samples with Gleason score 4–7 and 20 PCa samples with Gleason score 8–9. The ordinates represent the mean±s.e. (reported also in the inset). The increase in hnRNP K expression was found to be significant between NT and PCa with Gleason score 4–7 (*P*=0.014) and highly significant between Gleason score 4–7 and 8–9 (*P*=0.008). (**C**) Scatterplots of relative amounts of hnRNP K within the NM isolated from NT and PCa samples. Horizontal lines indicate the mean values. (**D**) Biochemical (PSA) progression-free survival according to hnRNP K expression within the NM. The dotted curve denotes patients (*n*=31) whose tumours had low hnRNP K expression (⩽0.60) and the solid curve denotes those (*n*=16) with high expression (>0.60); *P*=0.032.

Based on the hypothesis that the changes in the levels of hnRNP K would be more significantly associated with the NM rather than the whole cell, we focused our research on the quantification of hnRNP K expression within the NM, by 1D immunoblot analysis. Representative results are reported in Figure 2A, where the immunoblot patterns of hnRNP K isolated from NHP, NT and PCa, with different Gleason scores are compared. The use of the anti-hnRNP K antibody (see Materials and Methods) results in a highly specific recognition of both hnRNP K and the splicing variant previously designated hnRNP J (52 and 50 kDa, respectively) (Bomsztyk *et al*, 2004). The relative amounts of hnRNP K that remain associated with the NM are shown in Figure 2B, where the tumours were grouped into low-intermediate (Gleason score⩽7) and high-grade (Gleason score>7) lesions. HnRNP K undergoes progressive increase from NHP to NT to PCa; the differences in expression were significant between NT and PCa (*P*=0.0003), between NT and PCa with low-intermediate Gleason scores (*P*=0.014) and highly significant between low-intermediate and high Gleason scores (*P*=0.008). When we compared the expression of hnRNP K associated with the NM isolated from PCa with a Gleason score 4–6 with those from PCa with a Gleason score 7–9 the significance disappeared (*P*=0.1); however, if we separate Gleason 7 category group into two groups (3+4) and (4+3) according to primary differentiation pattern and compare the expression of hnRNP K in PCa with Gleason score 4–7(3+4) *vs* 7(4+3)–9 the difference becomes again statistically significant (*P*=0.017). This indicates that the level of hnRNP K associated with the NM does not only increase in tumour tissues, but also depends on the degree of differentiation.

The individual expression values of hnRNP K within the NM in 47 patients have been correlated with clinicopathological variables (Table 2). The protein expression was strongly correlated with the involvement of seminal vesicles and, as we have just mentioned, with the Gleason score. The average expression value of hnRNP K increased by 3.5 times in PCa (0.60, 95% CI 0.43–0.78) with respect to NT (0.17, 95% CI 0.08–0.27) (*P*=0.002) (Figure 2C). Using the average value in PCa, which corresponds to the 66.6th percentile, we have divided the patients into two groups: those with high expression of hnRNP K (>0.60, 16 patients) and those with low expression (⩽0.60, 31 patients). As reported in the Materials and Methods section, after a median follow-up time of 49.9 months (95% CI 20.4–80), 15 patients were found to have experienced biochemical progression. Patients who showed a good prognosis expressed low levels of hnRNP K, whereas those who showed the worst outcome expressed high levels of the protein (hazard ratio (HR) 2.95, 95% CI 1.05–8.29, *P*=0.032) (Figure 2D).

Is there a relationship between the IHC and WB results? To answer this question, we stratified the patients into four groups, based on the hnRNP K expression level within the NM (determined by WB) and in the cell as a whole or in the cytoplasm only (determined by IHC). Patients whose tumours expressed high levels of protein both within the NM and in the whole cell showed the worst outcome. Interestingly, these patients also had high levels of hnRNP K expression in the cytoplasm (cytoplasm score⩾4) (Table 3). Therefore, high levels of hnRNP K within the NM and in the cytoplasm, at the same time, are correlated with a higher risk of biochemical progression.

### Evaluation of hnRNP K phosphorylation state

**Figure 3:**
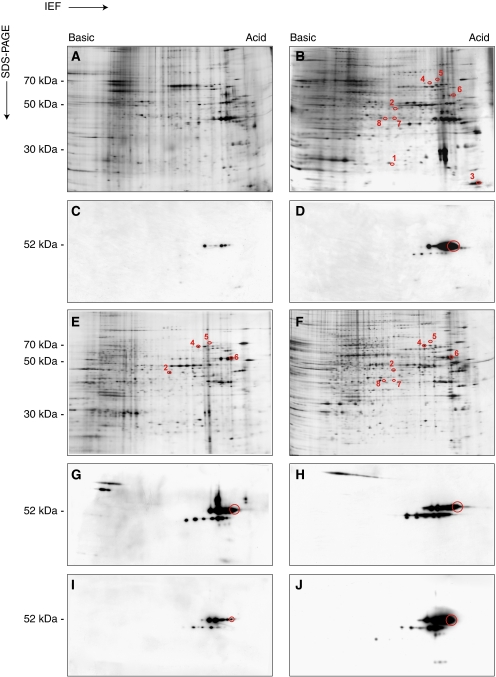
Representative 2D gel maps of the NM proteins extracted from NT (**A**) and PCa (**B**) tissues and from LNCaP (**E**) and PC3 (**F**) cell lines. Tumour-associated proteins are marked by circles and identified with the same number used in Boccardo *et al* (2003); silver-stained gels. The proteins resolved in 2D were transferred to Hybond-P membranes and probed with anti-hnRNP K antibody. Representative 2D WB of NM proteins extracted from NT (**C**), PCa (**D**), LNCaP and PC3 cells in the absence (**G** and **H**, respectively) and presence of 10 *μ*M BIC for 72 h (**I** and **J**, respectively). The circles in **D**, **G**, **H**, **I** and **J** highlight the NM-6 spot that corresponds to an acidic isoform of hnRNP K. This isoform is absent in NT (**C**) and is downexpressed in LNCaP cells after treatment with BIC (**I**). The relative molecular weights of standard proteins are reported on the left.

HnRNP K possesses multiple sites that are inducibly phosphorylated; this post-translational modification alters the interaction between the protein and nucleic acids or other proteins (Ostrowski *et al*, 2000). Moreover, cytoplasmic accumulation of hnRNP K is phosphorylation-dependent (Habelhah *et al*, 2001). Therefore, we carried out a preliminary characterisation of the changes in the phosphorylation state of hnRNP K by combining high-resolution 2D-PAGE with immunoblot analysis. NM proteins isolated from NT or PCa samples were separated and the gels were either stained with silver nitrate (Figure 3A and B) or transferred to the membrane and analysed by WB using the monoclonal antibody against hnRNP K (Figure 3C and D). Several spots were observed with molecular weight of 52 kDa and pI from 5.1 to 5.3 and with molecular weight 50 kDa and pI from 5.4 to 5.2, corresponding to hnRNP K and the isoform J, respectively. The global expression of hnRNP K was significantly higher in PCa compared with NT, and in particular the most acidic isoforms with molecular weight 52 kDa underwent significant increase. The comparison between auto-radiographic films and 2D maps confirm that the tumour-specific spot NM-6 corresponds to hnRNP K and it is an acidic isoform with pI 5.1. This result explains why in 2D-PAGE the NM-6 spot was present only in PCa and absent in both NHP and in NT. On the contrary, in 1D-PAGE and immunohistochemical experiments, even if in minor quantities, hnRNP K was also present in NHP and NT.

### Expression and modulation of hnRNP K in cell lines

**Figure 4:**
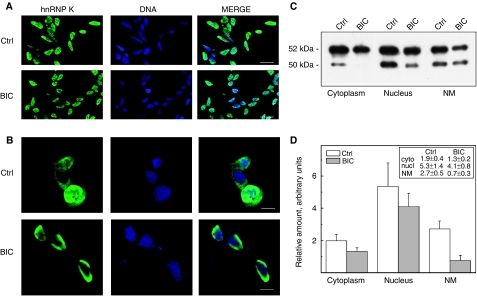
(**A, B**) Confocal microscopy analyses carried out on control LNCaP cells clearly show strong hnRNP K staining in the nucleus and lower staining in the cytoplasm. After exposure to 10 *μ*M BIC for 72 h, the staining of the cytoplasm disappears and the staining in the nucleus gets fainter. Cells were immunostained with anti-hnRNP K antibody (green) and DNA was visualised with SYTOX Orange (blue). Images from control (Ctrl) and treated (BIC) cells were acquired with identical acquisition settings and representative cells were photographed at low magnification (**A**; scale bars: 25 *μ*m) and at high magnification (**B**; scale bars: 10 *μ*m). (**C, D**) Comparison between hnRNP K expression, in the different cellular compartments, in control and treated cells. Representative 1D WB (**C**); the relative molecular weights are indicated on the left. The ordinates in (**D**) represent the mean±s.e. (reported also in the inset) of the relative amounts of hnRNP K, as determined by quantitative analysis, of four BIC treatment experiments and from four to seven different western blots, as in some samples were run on two different gels. When the cells were exposed to BIC, the expression of hnRNP K underwent an overall decrease. This decrease was significant within the NM (*P*=0.019).

The expression profile of NM proteins extracted from PCa tissue was compared with that of hormone-dependent (LNCaP) and hormone-independent (PC3) prostate carcinoma cell lines. Representative silver-stained gel patterns are reported in Figure 3E and F. More than 70% of the proteins expressed by the cell lines were common to PCa; it is interesting to note that six of the eight cancer-specific proteins, which we have previously found in PCa tissues (Alberti *et al*, 2000; Boccardo *et al*, 2003), were also expressed in the cell lines. In particular, NM-2, NM-4, NM-5 and NM-6 were expressed in both cell lines, whereas NM-7 and NM-8 were expressed only in hormone-insensitive cells. Therefore, these cell lines are good models to study the role of NM proteins after drug treatment. It must be pointed out that, in this work, we have studied only the expression of NM-6, which corresponds to an acidic isoform of hnRNP K as reported above. Both cell lines were treated for 72 h with 10 *μ*M BIC, a non-steroidal anti-androgen currently used as mono-therapy for locally advanced or biochemically recurrent PCa. Under these conditions, the maximum inhibitory effect on cell growth was observed (Boccardo *et al*, 2005). Representative 2D immunoblot experiments are shown in Figure 3. The protein was expressed both in LNCaP and PC3 cells and several spots, corresponding to different isoforms, were detectable (Figure 3G and H). After treatment with BIC, the expression of hnRNP K in LNCaP cells was remarkably decreased whereas PC3 cells were unaffected (Figure 3I and J). The expression of hnRNP K was also evaluated in LNCaP cells by confocal microscopy and 1D WB. In Figure 4A and B, representative optical sections are reported. In untreated cells, hnRNP K was above all localised in the nucleoplasm; however, a little fluorescence intensity was also present in the cytoplasm; moreover, as it is well-known, the protein is preferentially concentrated in the discrete loci referred to as splicing factor compartments (Dundr and Misteli, 2001). After treatment with BIC, hnRNP K was absent in the cytoplasm and present in a minor quantity in the nucleus, where it tended to move towards the periphery. Quantitation of hnRNP K expression, carried out by 1D WB, confirmed that the protein was present in all subcellular compartments (cytoplasm, nucleus and NM) (Figure 4C and D) and when the cells were exposed to BIC, the expression of hnRNP K underwent an overall decrease predominantly within the NM (from 2.72±0.49 to 0.75±0.34, *P*=0.019).

## Discussion

In this study, for the first time, we have provided clear evidence that hnRNP K is significantly overexpressed in PCa with respect to NT tissue; moreover, an acidic isoform of this protein is exclusively present in tumour cells. Higher expression within the NM and aberrant cytoplasmic localisation were significantly associated with poor prognosis. Preliminary results indicate that hnRNP K is negatively modulated *in vitro* by the exposure to a non-steroidal anti-androgen and therefore that it probably occurs as a result of androgen stimulation.

The correlations between elevated expression of hnRNP K and tumour development and progression are well documented (Carpenter *et al*, 2006a) and the results reported here are in line with those findings. We have demonstrated that, in PCa, this increase is more evident within the hnRNP K fraction that is part of NM. The NM is the protein scaffold that provides the structural framework for organising chromatin and plays a pivotal role in the spatial and temporal coordination of gene activation events (Zink *et al*, 2004). The hnRNPs are among the most abundant components of the NM. The interactions of chromatin with the NM occur by specific DNA sequences called MARs (Matrix Attachment Regions), which are placed close to the transcriptionally active regions of chromatin so that transcription is initiated in regions of chromatin anchored to the NM. We have demonstrated that several proteins belonging to the hnRNP family bind DNA-MARs (Barboro *et al*, 2009) and it is known that hnRNP K binds both RNA and DNA (Bomsztyk *et al*, 1997). In addition, our preliminary experiments (data not shown) indicate that, in the prostate model, hnRNP K also binds MAR sequences. Therefore, the increased level of hnRNP K in the NM could be the critical step towards the activation of the numerous pathways involved in carcinogenesis, which are potentially regulated by hnRNP K. HnRNP K could directly or indirectly, through modifications of protein/protein or RNA complexes, modulate the transition from an open to a condensed chromatin state. This results in the passage from a transcriptionally competent to an incompetent state, which in turn determines the modification of the expression of several genes. Furthermore, it has been demonstrated that during differentiation the splicing factor compartments, which contain hnRNPs, are relocated from internal regions towards the periphery of the nucleus (Dundr and Misteli, 2001). We observed that, after treatment with BIC, not only does the expression of hnRNP K decrease but the protein was invariably found at the nuclear periphery. The interaction between hnRNP K and nucleic acids and/or proteins could be regulated by the state of phosphorylation of the protein.

Cytoplasmic localisation of hnRNP K is phosphorylation-dependent and is carried out by mitogen-activated protein kinase/extracellular signal-regulated kinase that phoshorylates the protein both *in vivo* and *in vitro* at Ser^284^ and Ser^353^ (Bomsztyk *et al*, 2004). The phosphorylation may occur primarily in the nucleus and afterwards the protein may be translocated to the cytoplasm. Therefore, phosphorylated hnRNP K may modify the nuclear-shuttling domain and facilitate its cytoplasmic accumulation (Habelhah *et al*, 2001). The cytoplasmic accumulation of hnRNP K seems to be an intrinsic feature of transformation *per se*, and does not merely reflect the increase in cell proliferation. In fact Ostrowski and Bomsztyk (2003) reported that levels of nuclear hnRNP K were higher in proliferating hepatocytes compared with resting cells, but levels of cytoplasmic hnRNP K were the same or lower in dividing compared with quiescent cells. An important role has been suggested for cytoplasmic hnRNP K in nasopharyngeal carcinoma and metastasis, where aberrant cytoplasmic localisation of hnRNP K along with overexpression of thymidine phosphorylase is associated with shorter overall survival and lower metastasis-free survival (Chen *et al*, 2008). Interestingly, in a loss-of-function screening system based on intracellular expression of single-domain antibodies, Inoue *et al* (2007) found that hnRNP K is a potential target for metastasis and its cytoplasmic accumulation may mediate its role in cell migration and metastasis. Our findings, namely the overexpression of an acidic isoform in PCas and the association between the hnRNP K cytoplasmic accumulation with poor prognosis, are in line with the above results and further support the possible role for cytoplasmic hnRNP K in tumour aggressiveness.

We have shown here that after treatment with BIC, the expression of hnRNP K in LNCaP cells underwent an overall decrease predominantly within the NM. Additional studies will determine whether this is a specific effect of the anti-androgen used here or simply it represents the result of silencing of the gene, which encodes for this protein under androgen stimulation. Whatever the mechanism involved, hnRNP K might play a role in the regulation of AR gene. Wang *et al* (2008) showed that in LNCaP cells 5′-untranslated region of the AR promoter harbours a suppressor element, which is directly bound hnRNP and Purα, but only the regulation of this latter is crucial for the repression of AR levels. Vice versa Shi *et al* (2008) have reported that transcriptional downregulation of AR in the aging rat liver and in oxidatively stressed hepatoma cells involve hnRNP K through the interactions with PARP-1 and the first may serve as a stabilising docking platform for the activating complex that governs AR stimulation; in this model, silencing of hnRNP K decreased AR expression. Moreover, we were able to show, utilising the same approach, that the knockdown of hnRNP K expression gives rise to a loss of the angiogenic and migratory phenotype of prostate carcinoma cells (Benelli *et al*, 2009). Therefore this protein could provide both a novel therapeutic strategy to control PCa progression and metastasis and check the answer to hormonal therapy.

Our data, therefore, confirm previous results and support the hypothesis of Carpenter and Murray (2007) that this protein probably plays a key role in the carcinogenesis process and could also be a useful biomarker for disease diagnosis, progression and prognosis. HnRNP K might also represent a new therapeutic target. Work is in progress in our laboratory to produce an antibody specific for acidic isoform present only in PCa, which might significantly improve upon the sensitivity and specificity with which PCa is diagnosed and could help to stratify patients into different prognostic subgroups.

## Figures and Tables

**Table 1:** Patient demographics and tumour characteristics

	***N*=49**	**(%)**
Median preoperative age, years (range)	64.0 (48.0–77.0)	
Median preoperative PSA, ng ml^–1^ (range)	11.0 (5.0–120.0)	
		
*Tumour stage*		
pT2	24	(49.0)
pT3	24	(49.0)
pT4a	1	(2.0)
		
*Pelvic nodes involved*		
pN0	26	(53.1)
pN1-2	10	(20.4)
pNx	13	(26.5)
		
Surgical margins involved	20	(40.8)
Seminal vesicles involved	12	(24.5)
		
*Gleason score*		
⩽6	16	(32.7)
=7	12	(24.5)
>7	21	(42.9)

**Table 2:** Significance of the relationship between the relative amount of hnRNP K within the NM and patient characteristics

**Patient characteristics**	**Pearson's correlation coefficient**	***P-*value**
Preoperative age	0.102	0.5
Preoperative PSA	0.052	0.7
Capsular penetration	−0.087	0.6
Surgical margins involved	0.264	0.07
Seminal vesicles involved	0.330	0.02
Gleason score >7	0.381	0.008
Pelvic nodes involved	0.252	0.08

**Table 3:** Ability to predict biochemical progression according to hnRNP K expression in the different cellular compartments

**hnRNP K expression**	***n***	**obs^a^**	**HR (95% CI)**	***P-*value**
*Within the NM and the whole cell* b				
⩽0.60 and <10	16	2	1.0	0.1
⩽0.60 and ⩾10	14	6	3.96 (0.79–19.86)	0.1
>0.60 and <10	7	3	5.03 (0.83–30.51)	0.08
>0.60 and ⩾10	9	4	9.49 (1.61–55.82)	0.013
				
*Within the NM and the cytoplasm*				
⩽0.60 and <4	12	2	1.0	0.1
⩽0.60 and ⩾4	18	6	2.41 (0.48–12.03)	0.3
>0.60 and <4	7	3	3.89 (0.64–23.67)	0.1
>0.60 and ⩾4	9	4	7.34 (1.25–43.13)	0.025

a obs (observed)=number of patients with a biochemical progression.

b hnRNP K expression level was determined within the NM by WB and in whole cell or in cytoplasm by IHC.
